# Refractory Autoimmune Hemophagocytic Lymphohistiocytosis in Adults: Multi-therapy Failure and the Limits of Salvage Transplantation

**DOI:** 10.7759/cureus.106267

**Published:** 2026-04-01

**Authors:** Bsher Almaalouli, Hannah Smith, Gabriel R Kirsch, Vincent Courant, Gurjit S Kaeley

**Affiliations:** 1 Internal Medicine, University of Central Florida/North Florida Hospital, Gainesville, USA; 2 Rheumatology, University of Florida College of Medicine – Jacksonville, Jacksonville, USA; 3 Internal Medicine/Rheumatology, University of Florida College of Medicine – Jacksonville, Jacksonville, USA

**Keywords:** biologic agents, cytokine release storm, hematopoietic stem cell transplantation (hsct), hemophagocytic lymphohistiocytosis (hlh), immunosuppressive therapy in sle, macrophage activation syndrome (mas), multiorgan system failure, systemic lupus erythematosus (sle)

## Abstract

This article presents a case of refractory autoimmune-associated hemophagocytic lymphohistiocytosis (HLH) complicating systemic lupus erythematosus in a young adult. It highlights the diagnostic difficulty created by overlapping features of infection, autoimmune flare, and cytokine-driven hyperinflammation, and details disease progression despite corticosteroids, etoposide, multiple biologic agents, and consideration of hematopoietic stem cell transplantation (HSCT). The discussion broadens to review the epidemiology of autoimmune-triggered HLH in adults, reported response rates to cytokine-directed therapies, the limited role and toxicity of salvage cytotoxic regimens, and contemporary HSCT outcomes, which demonstrate variable survival but remain the only potentially curative strategy in selected patients.

Clinicians managing patients with severe or treatment-resistant autoimmune disease should maintain a low threshold for suspecting HLH when cytopenias, hyperferritinemia, or unexplained organ dysfunction emerge. Early calculation of the HScore and prompt initiation of therapy are critical to reducing diagnostic delay. While definitive management requires control of the underlying immune dysregulation, treatment of active hyperinflammation should not be postponed during ongoing evaluation and multidisciplinary planning.

## Introduction

Hemophagocytic lymphohistiocytosis (HLH) is a potentially fatal disease of pathologic immune response. In adults, secondary HLH is most commonly precipitated by malignancy, infection, or autoimmune disease, the latter often referred to as macrophage activation syndrome (MAS) [1]. Despite high-dose corticosteroid-based therapy and etoposide-based regimens, a sizeable percentage of patients reach a refractory disease, and the disease is incurable with mortality rates over 60% [2]. This therapeutic approach pursues the use of subsequent biologic agents and finally, hematopoietic stem cell transplantation (HSCT) as a possibly definitive intervention. We highlight a case of a 21-year-old patient who was refractory to first-line HLH therapies and did not respond to targeted immunotherapies [2,3]. Alongside this case, a dedicated narrative review, which quantifies the burden of refractory autoimmune HLH in adults, critically analyzes the evidence utilized in salvage therapies, and presents a data analysis of the role, results, and limitations of HSCT in this patient group.

## Case presentation

We report a case of difficult-to-treat systemic lupus erythematosus (SLE) in a 21-year-old female with a prolonged hospital course, marked by cerebritis and nephritis, who presented with new-onset generalized tonic-clonic seizures. She had been hospitalized multiple times over the past few years for lupus nephritis and altered mental status, most recently for cerebritis requiring IV cyclophosphamide, which was complicated by the development of posterior reversible encephalopathy syndrome (PRES).

Her home medications included prednisone 30 mg daily, mycophenolate mofetil 500 mg twice daily, and hydroxychloroquine 200 mg daily.

**Figure 1:**
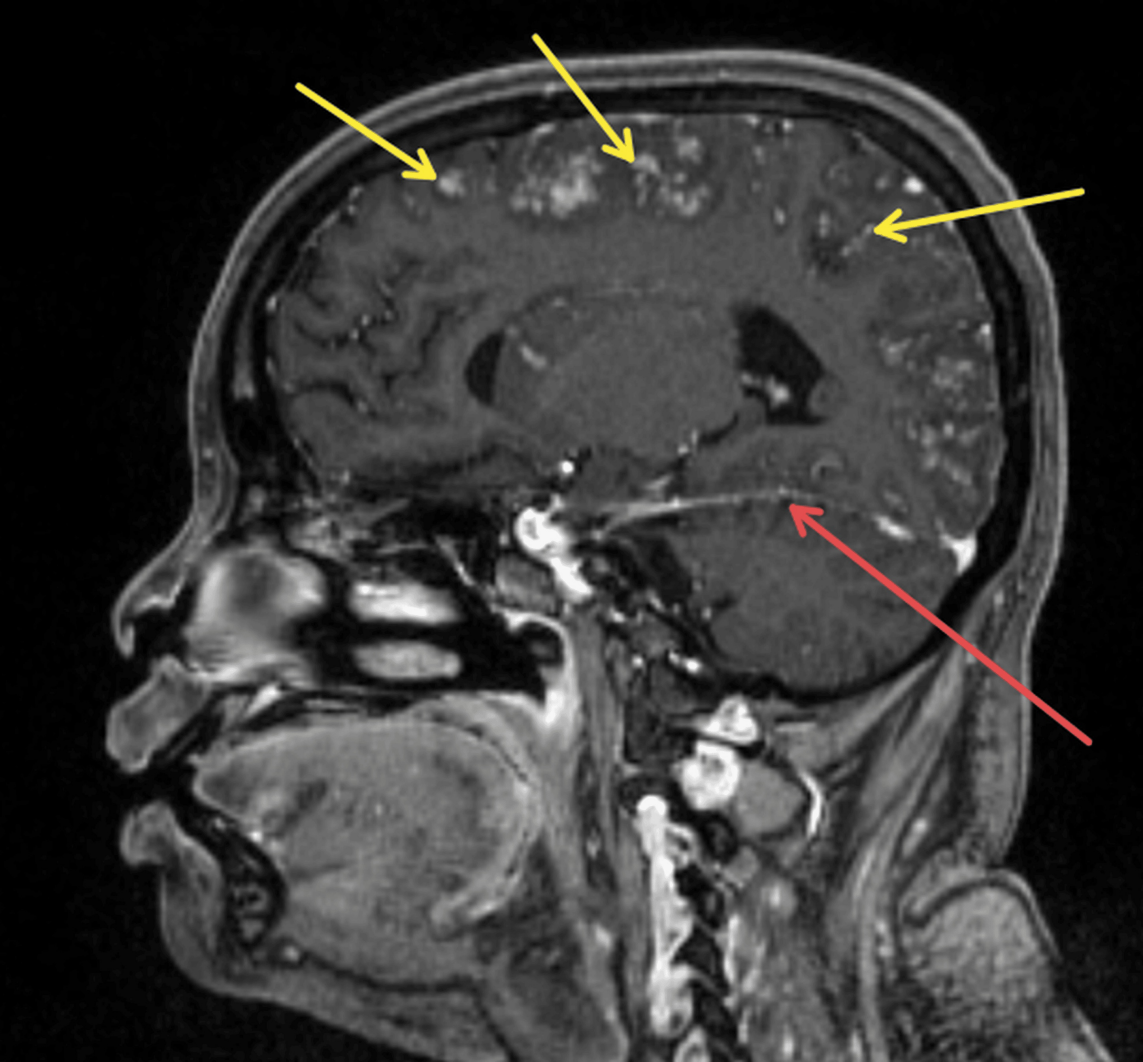
Sagittal post-contrast T1-weighted MRI demonstrating multiple nodular enhancing hyperintensities (yellow arrows), along with leptomeningeal enhancement (red arrow).

**Figure 2:**
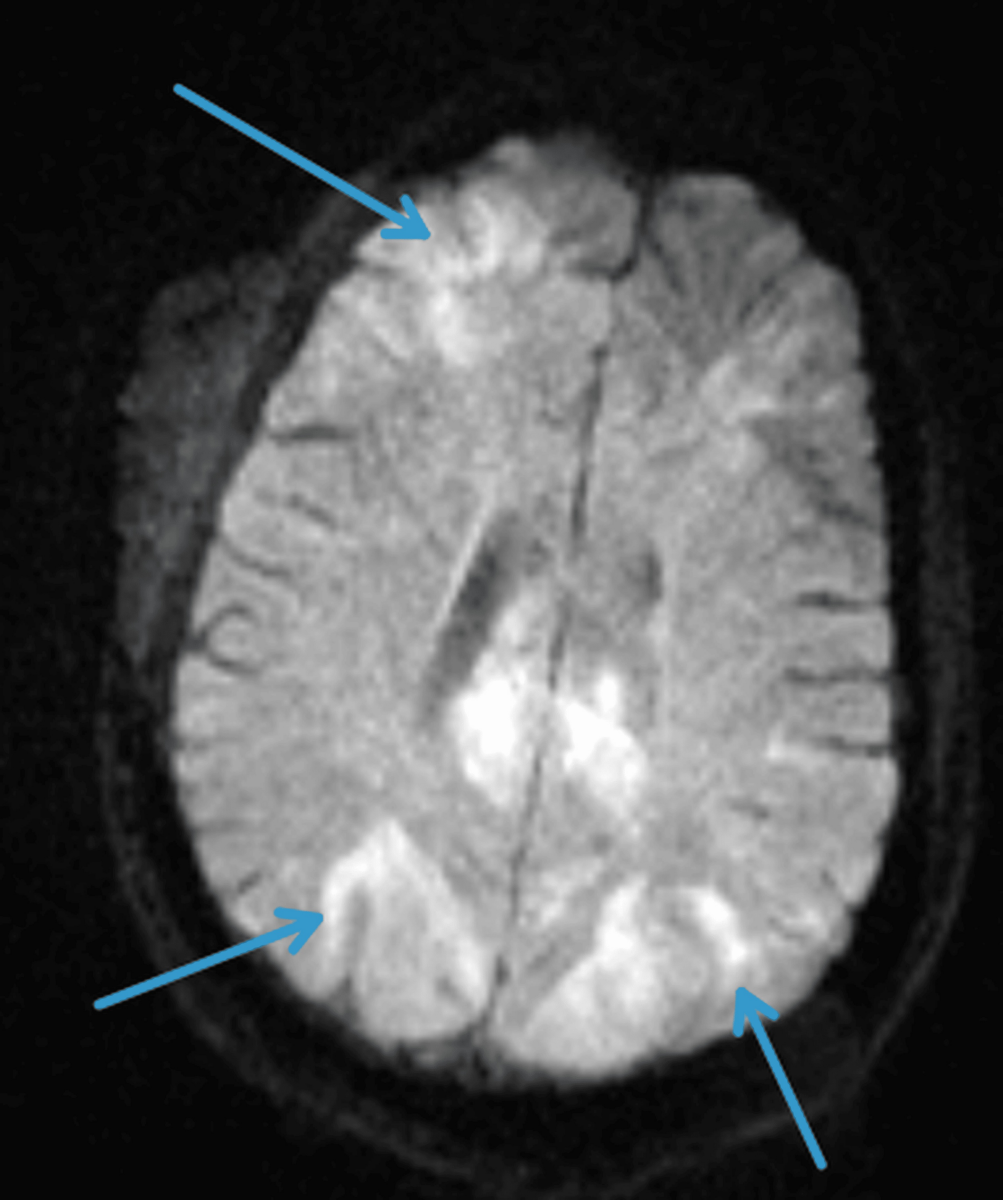
Axial diffusion-weighted MRI demonstrating multifocal areas of diffusion restriction consistent with cytotoxic edema and active inflammatory involvement.

On admission, she was febrile to 102.4°F (39°C) and encephalopathic. Laboratory evaluation revealed pancytopenia, hyperferritinemia, elevated liver enzymes, and fibrinogen (Table 1). Her HScore was 230, consistent with a high probability of HLH. Brain MRI showed diffuse nodular leptomeningeal enhancement with extensive cortical and subcortical hyperintensities bilaterally (Figures 1, 2). Cerebrospinal fluid analysis was unremarkable, including testing for Epstein-Barr virus (EBV), cytomegalovirus (CMV), and JC virus PCR. Evaluation for fungal and bacterial infections was also negative.

**Table 1:** Initial laboratory findings at presentation.

Test	Result	Reference range	Unit
White blood cells (WBC)	0.89	4.0-11.0	×10⁹/L
Hemoglobin	8.2	12-16 (female), 13-17 (male)	g/dL
Platelet count	92	150-450	×10⁹/L
Ferritin	14,000	30-400	ng/mL
Triglycerides	606	<150	mg/dL
Aspartate aminotransferase (AST)	93	10-40	U/L
Alanine aminotransferase (ALT)	46	7-56	U/L
Lactate	3.3	0.5-2.2	mmol/L
Fibrinogen	477	200-400	mg/dL
Urinalysis	>300 protein	Negative	-
Antinuclear antibody (ANA)	1:640 (Positive)	Negative (<1:80)	Titer
Anti-double-stranded DNA (Anti-dsDNA)	5	0-9	IU/mL
Complement C3	75	90-180	mg/dL
Complement C4	23	10-40	mg/dL
Erythrocyte sedimentation rate (ESR)	87	0-20	mm/hr

She was initially treated with high-dose IV methylprednisolone 1,000 mg daily (hospital days one to four), then transitioned to 1 mg/kg daily. Given her thrombocytopenia and recent gastrointestinal illness with imaging evidence of enterocolitis and mesenteric edema, plasma exchange was initiated out of concern for possible thrombotic microangiopathy (thrombotic thrombocytopenic purpura/hemolytic uremic syndrome). Although mental status and organ function improved briefly, she developed persistent pancytopenia and recurrent fevers.

A bone marrow biopsy revealed hemophagocytic histiocytes without evidence of dysplasia or malignancy. Soluble IL-2 receptor levels were mildly elevated (970 U/mL; reference range typically <600 U/mL). Given the constellation of hyperferritinemia, hypertriglyceridemia, cytopenias, and hemophagocytosis, she was diagnosed with acquired HLH in the setting of active SLE.

She was started on subcutaneous anakinra, which was discontinued after three days due to lack of clinical response, suspected due to poor absorption in the setting of subcutaneous edema. Etoposide was initiated but discontinued after the second dose due to worsening thrombocytopenia and myelosuppression. Subsequently, she received two doses of emapalumab, with the first being 1 mg/kg, administered with IV anakinra. As her ferritin levels and HLH activity continued to worsen, she developed acute chest pain and hypoxia, and echocardiography revealed a large hemorrhagic pericardial effusion, requiring pericardiocentesis. In an effort to control persistent inflammation, ruxolitinib was initiated on hospital day 38 but was discontinued due to progressive thrombocytopenia. Rituximab and intravenous immunoglobulin (IVIG) were subsequently introduced to treat refractory HLH and warm autoimmune hemolytic anemia. Cyclosporine was later trialed for persistent HLH and proteinuria but was discontinued due to nephrotoxicity and recurrence of PRES.

**Figure 3:**
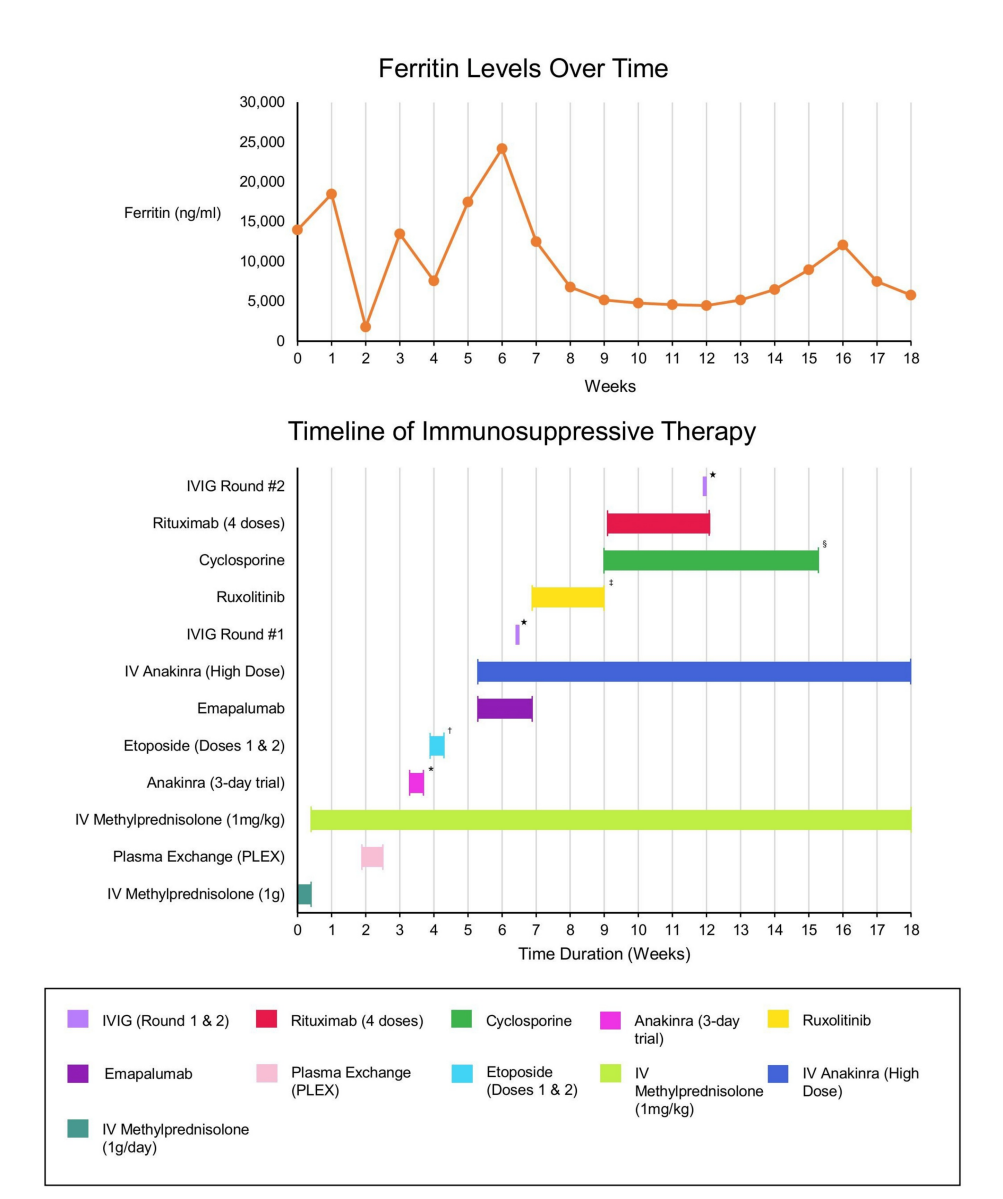
Ferritin levels during the hospital course and corresponding immunosuppressive therapies. Discontinuation notes: * Initial anakinra trial discontinued due to lack of clinical response. † Planned additional etoposide dosing withheld due to thrombocytopenia. ‡ Ruxolitinib discontinued because of worsening thrombocytopenia. § Cyclosporine discontinued following magnetic resonance imaging findings suggestive of posterior reversible encephalopathy syndrome (PRES). ★ denotes acute interventions (e.g., IVIG at weeks 6.4 and 11.9). IVIG: intravenous immunoglobulin; PLEX: plasma exchange.

Despite sequential and overlapping therapies, the patient remained unresponsive to therapy. She experienced frequent relapses with any attempt to taper steroids, and her ferritin levels and cytopenias fluctuated but remained persistently abnormal (Figure 3).

Her hospital course progressed with escalating complications related to uncontrolled hyperinflammation. Early in the course, she developed recurrent PRES and persistent cytopenias, followed by acute hypoxic respiratory failure requiring intubation and recurrent pulmonary edema. She subsequently developed upper extremity deep vein thrombosis and proteinuric acute kidney injury requiring dialysis. Later, her course was complicated by gastrointestinal bleeding and the development of a large hemorrhagic pericardial effusion requiring pericardiocentesis.

By the third month of hospitalization, she continued to have persistent cytopenias and markedly elevated ferritin levels, consistent with refractory HLH, while still requiring high-dose corticosteroids and anakinra to control inflammation. Despite multiple lines of immunosuppressive therapy, there was no durable clinical improvement. HSCT was considered but declined by multiple tertiary centers due to her poor condition, multiorgan failure, and anticipated high risk of mortality associated with the induction regimen. These factors prompted ongoing multidisciplinary discussions about goals of care. At the time of writing, the patient had elected to pursue hospice care in light of her overall prognosis.

## Discussion

Narrative review: Quantifying the challenge and evaluating the last resort in adult refractory autoimmune HLH

Epidemiology, Pathophysiology, and Defining the Refractory State

Autoimmune diseases are responsible for about 15‑20% of the cases of adult secondary HLH. The estimated incidence of autoimmune-associated HLH is 0.2‑0.3 per 100,000 adults per year [4,5]. The pathophysiology involves a positive feedback loop in which there is impaired NK/T‑cell cytotoxicity that fails to kill antigen-presenting cells, resulting in excess production of interferon‑gamma, IL-1‑beta, IL-6, IL‑18, and tissue necrosis factor‑alpha [6]. In the case of SLE, this can be triggered by flares, infections, or medications. Standard first-line therapy involves high-dose corticosteroids (e.g., methylprednisolone 1‑2 mg/kg/day or pulse therapy) with initial responses in 40‑60% of cases. Severe or steroid-refractory disease is usually treated with the etoposide and dexamethasone-based HLH‑94 protocol [7]. But primary refractoriness, or the inability to respond with at least a partial response in two to three weeks of first-line treatment, is seen in 20‑35% of patients with autoimmune HLH as adults [8,9]. Medically refractory disease, when treatment occurs despite corticosteroids and at least one second-line agent (e.g., etoposide and cyclosporine), is described with a mortality rate of 60‑80% in modern series [10,11].

In our case, the patient presented with pancytopenia, severe hyperferritinemia, hypertriglyceridemia, and a high HScore consistent with HLH. Similar to previously reported SLE-associated HLH cases, diagnosis was complicated by overlapping features of active lupus and systemic inflammation. Despite early corticosteroid therapy and etoposide, the patient failed to achieve disease control, representing primary refractory autoimmune-associated HLH, a subgroup known to have particularly poor outcomes.

The Biologic Salvage Ladder and the Reality of Multi-agent Failure

The treatment landscape has changed with the introduction of cytokine-targeted therapies. Anakinra (IL-1 receptor antagonist) has been found to be highly effective in adult-onset Still’s disease-associated macrophage activation syndrome (AOSD-MAS), with response rates in retrospective studies ranging from 70% to 85% [12]. However, in non-AOSD autoimmune HLH (e.g., SLE), response rates are less encouraging, ranging between 50% and 60% [13]. Etoposide is also for severe or refractory MAS-HLH, specifically for patients with severe disease or CNS involvement who fail glucocorticoids, cyclosporine, and/or anakinra. Tocilizumab (IL-6R inhibitor) has shown efficacy in case series, but its use can be complicated by transaminitis and opportunistic infections [14]. The Janus kinase (JAK) inhibitor ruxolitinib, by blocking signaling downstream of multiple cytokines, has been shown to be promising. A pilot trial of secondary HLH reported an overall response rate of 65% at four weeks, but complete remission rates were lower [15].

Despite these options, multi-refractory disease, which is failure of etoposide and two or more targeted biologics, is associated with a uniformly poor prognosis with mortality approaching 90% in some series [16]. This refractoriness may be due to redundant pathways of cytokines, irreversible damage to end organs, or underlying genetic predispositions. Up to 15-20% of adults with apparent "secondary" HLH have heterozygous or hypomorphic mutations in the familial HLH genes (PRF1, UNC13D, STXBP2), which could be predictive of a more severe course and poor response to conventional immunosuppression [17].

When targeted therapies have failed, salvage cytotoxic regimens are considered. The DEP (doxorubicin, etoposide, methylprednisolone) regimen had a response rate of 89.5% in a Chinese study of adult refractory HLH but was associated with significant hematologic and infectious toxicity [18]. Alemtuzumab (anti-CD52) and antithymocyte globulin induce profound lymphodepletion and have response rates of 40-60%, but at the expense of high rates of CMV reactivation and other opportunistic infections [19].

Our patient exemplifies this treatment-refractory phenotype. She received sequential therapies without a durable clinical response. Treatment intolerance further limited options, as cytopenias and drug-related complications necessitated discontinuation of several agents. This course highlights the clinical challenge of managing HLH when both disease activity and treatment toxicity progressively restrict available therapies.

Hematopoietic Stem Cell Transplantation: Utilization, Indications, and Outcomes

Allogeneic HSCT is the only potentially curative strategy as it replaces the dysregulated immune system. Its use in the case of adult secondary HLH is rare and is not standardized.

Utilization rates and indications: HSCT is used in <5% of all cases of secondary HLH [20]. In the largest systematic review so far (2019), only 38 adult cases of HSCT for secondary HLH could be found, of which 11 (29%) had an autoimmune/rheumatic trigger [21]. Common pragmatic indications are (1) persistent, active HLH requiring continuous high-intensity immunosuppression, (2) recurrent life-threatening flares upon tapering therapy, (3) identification of a pathogenic genetic variant, and (4) development of treatment-related complications (e.g., therapy-related myeloid).

Transplant outcomes - a data synthesis: Outcomes of allogeneic HSCT for adult refractory secondary HLH are reported in Table 2.

**Table 2:** Reported outcomes of allogeneic HSCT for adult refractory secondary HLH (selective series). * Initial anakinra trial discontinued due to lack of clinical response. HSCT: hematopoietic stem cell transplantation; HLH: hemophagocytic lymphohistiocytosis; OS: overall survival; RIC: reduced-intensity conditioning; NRM: non-relapse mortality; TRM: transplant-related mortality; EBMT: European Society for Blood and Marrow Transplantation; CMWP: Chronic Malignancies Working Party; IEWP: Inborn Errors Working Party.

Study (year)	Population (n)	Autoimmune subset (n)	Key findings (autoimmune/overall cohort)
Gooptu et al. (2023) [22]	Adult HLH (primary + secondary), transplanted (n = 21)*	Not separately specified	3-year OS ~75% with RIC + alemtuzumab; relapse ~15%; non-relapse mortality ~15% at 3 years
Machowicz et al. (2022) (EBMT CMWP/IEWP registry) [23]	Adult HLH (≥18 years) transplanted (n = 87)	Not specified	3-year OS ~44%, 5-year OS ~44%; relapse ~21%; NRM ~36%
Kim et al. (2024) (Japanese HSCT registry) [24]	Adult primary/secondary HLH (n = 56)	Not specified	3-year OS ~40.6%; relapse ~19.8%; NRM ~39.6%
Yoon et al. (2019) [25]	Adult secondary HLH (non-malignant) transplanted (n = 14)	Not specified	Post-HSCT 2-year OS ~64-65%; TRM ~28-29% (better when disease is controlled pre-HSCT)
Masood et al. (2022) (Aggregate small case series/reviews) [26]	Mixed adult refractory sec-HLH (n ≈ <50)	Some autoimmune cases	Estimated OS ranges from ~40–75%; TRM/NRM is highly variable (15-40%); outcomes are worse with active disease at transplant

Interpreting Salvage HSCT in Refractory Autoimmune-Associated HLH

The transplant literature for refractory autoimmune-associated HLH in adults is also limited, heterogeneous, and mostly observational in nature, but several coherent interpretive themes emerge when the available series are reviewed together. Outcomes following HSCT differ strikingly in different cohorts, related to differences in patient selection, control of disease at the time of transplant, and to the transplant platform rather than inconsistency of effect alone. In highly selected single-center cohorts, modern reduced-intensity conditioning (RIC) in combination with early lymphodepletion has resulted in three-year overall survival approaching 75% with relapse rates approaching 15% and non-relapse mortality of 15% [22]. These results are probably the best-case situations that can be achieved under good circumstances and are not theorized to be normal expectations.

In comparison, the less conservative value of a real-world outcome is given by registry-based analyses. The European Society for Blood and Marrow Transplantation (EBMT) Chronic Malignancies Working Party (CMWP)/Inborn Errors Working Party (IEWP) registry reported three and five-year overall survival of about 44% in 87 transplanted adults with relapse rates of about 21% and non-relapse mortality of nearly 36% [23]. Comparable results were reported in the Japanese national transplant registry, where three-year survival was 40.6% and non-relapse mortality was close to 40% [24]. Smaller adult series are in an intermediate position. In a non-malignant secondary HLH cohort, two-year post-transplant survival was in the range of 64-65%, but transplant-related mortality was high at almost 30%, which highlights the frailty of this cohort [25,26].

Across all of the studies, there is one signal that is remarkably consistent. Pathological activity of the disease at the time of transplant is the leading prognostic factor. Patients who go on to HSCT with HLH in remission or with significant reduction of inflammation show lower rates of relapse and lower rates of non-relapse death than patients transplanted with active disease [22,23,25]. This association is biologically plausible, as uncontrolled cytokine-mediated inflammation adds to the damage of the liver, the endothelium, the bone marrow, and infectious susceptibility, and therefore multiplies the toxicity of conditioning. The implication is that it is clinically challenging. Patients with the most aggressive, refractory disease are the ones who can benefit most from immune replacement but are often the least able to tolerate transplantation. As a result, registry outcomes probably underestimate true population mortality since patients who die prior to referral or are considered ineligible for the registry are not identified.

These observations are informative for current practice and are limited. RIC regimens such as fludarabine-based combinations with busulfan or melphalan are currently preferred in adults to reduce organ toxicity, and there is no evidence that myeloablative conditioning offers any benefit to disease control (i.e., no convincing evidence of superiority of myeloablative conditioning over RIC regimens such as fludarabine-based combinations with busulfan or melphalan in adults in terms of control of disease) [22,23,27]. Nonetheless, there is no consensus about the best conditioning backbone or source of donor, or the best strategy for serotherapy. Relapses of HLH after HSCT are seen in about 10-20% of cases, while graft versus host disease is a major contributor to late morbidity and mortality in series [22-24].

Interpretation of these data is limited by basic limitations. Selection bias is extreme, with only a minority of patients with preserved organ function presenting for transplant. There are no randomized or strictly matched comparative studies that could prove the superiority of HSCT to continued medical therapy. Marked heterogeneity in autoimmune diagnoses, genetic background, and transplant approaches preclude meaningful subgroup analysis. Definitions of refractoriness and of adequate pre-transplant disease control are highly variable, and long-term outcomes beyond two to three years, chronic graft-versus-host disease (GVHD), quality of life, secondary malignancies, and post-transplant autoimmune disease activity are poorly characterized.

Overall, HSCT can be considered a risky but possibly disease-modifying therapy for a strictly limited group of refractory autoimmune-related adults with HLH, especially in cases when inflammation can be inhibited prior to conditioning. The existing evidence is in favor of careful and personalized application instead of an algorithmic one, and the necessity of harmonized definitions, future registries, and joint research that would reflect the entire denominator of patients in the care of transplant.

In our case, HSCT was considered as a potential salvage strategy after failure of multiple immunosuppressive agents. However, the patient’s deteriorating clinical status, including multiorgan failure, dialysis-dependent kidney injury, recurrent respiratory failure, and ongoing hyperinflammation, ultimately precluded transplant eligibility. This situation reflects a frequent real-world challenge: patients who may theoretically benefit from HSCT often become ineligible due to rapid disease progression and cumulative treatment toxicity.

Critical Limitations, Case Integration, and Clinical Implications

The existing evidence base to guide HSCT in adult autoimmune-associated HLH is limited by a number of structural deficiencies that limit confident clinical use of this procedure. Foremost is extreme selection prejudice. Published transplant cohorts are a highly selected subgroup with preserved performance status and organ reserve and do not represent patients who deteriorate rapidly, die during evaluation, and are ineligible for transplant, and thus inflate the perceived survival [27]. In parallel, there are no randomized or propensity-matched comparisons of HSCT with continued best medical therapy, so no definitive statement of survival benefit is possible [28]. Interpretation is also complicated by the fact that studies are markedly heterogeneous, combining different autoimmune diseases, genetic backgrounds, types of donors, and conditioning regimens, making it unreliable to conduct meaningful subgroup analyses [23,27]. Definitions of refractoriness and adequate pre-transplant disease control are inconsistent, and the follow-up is usually limited to two to three years so that long-term outcomes, including chronic GVHD, quality of life, secondary malignancies, and post-transplant autoimmune activity, remain poorly defined [29].

The case of multi-refractory SLE-associated HLH presented here illustrates such issues. Failure of high-dose corticosteroids and etoposide-based therapy and multiple targeted agents leaves such patients in a risk category where expected mortality is greater than 80% [16]. Against this backdrop, HSCT seems to be the only intervention with curative intent, but its success is closely linked with achieving inflammatory control before conditioning [22,23]. Sequential biologic therapies, although medically rational, consume a limited therapeutic window during which the organs may still be functioning sufficiently to allow for transplantation. This prompts a critical unresolved question: whether formal transplant evaluation should be activated earlier in the disease course, following failure of etoposide and one targeted agent, as opposed to deferred until exhaustion of all medical options [28,30].

Aggregated data indicate an approximately 58-65% post-HSCT three-year survival that is offset by treatment-related mortality of almost 30-35% [22,24]. This critical risk-benefit calculus requires individualized decision-making that integrates organ function, psychosocial context, and donor availability. Clinically, these data are supportive of earlier transplant consultation, goal-directed therapy (bridging therapy) targeted to achieve transplant readiness, and the development of international registries and predictive biomarkers to identify patients who are likely to develop multi-refractory disease and benefit from earlier curative intent strategies [27,29,30].

## Conclusions

Refractory autoimmune-associated HLH in adults carries a poor prognosis, as demonstrated in this severe case of SLE. HSCT is the sole potentially curative modality that has been subjected to delayed referral and cumulative procedural burden by the advanced disease and progressive toxicity with sequential salvage therapies. The evidence in the currently available literature, while limited and of low quality, indicates the possibility of durable remission in a subset of carefully selected patients. The key to success is to be able to control the cytokine storm before transplant conditioning starts. Moving forward, a more standardized, proactive, and collaborative approach between rheumatologists, hematologists, and transplant physicians is needed to achieve better outcomes in this vulnerable population.

## References

[1] Ramos-Casals M, Brito-Zerón P, López-Guillermo A, Khamashta MA, Bosch X (2014). Adult haemophagocytic syndrome. Lancet.

[2] Risma KA, Marsh RA (2019). Hemophagocytic lymphohistiocytosis: clinical presentations and diagnosis. J Allergy Clin Immunol Pract.

[3] Schram AM, Berliner N (2015). How I treat hemophagocytic lymphohistiocytosis in the adult patient. Blood.

[4] Li J, Wang Q, Zheng W, Ma J, Zhang W, Wang W, Tian X (2014). Hemophagocytic lymphohistiocytosis: clinical analysis of 103 adult patients. Medicine (Baltimore).

[5] Parikh SA, Kapoor P, Letendre L, Kumar S, Wolanskyj AP (2014). Prognostic factors and outcomes of adults with hemophagocytic lymphohistiocytosis. Mayo Clin Proc.

[6] Canna SW, Marsh RA (2020). Pediatric hemophagocytic lymphohistiocytosis. Blood.

[7] Lachmann G, La Rosée P, Schenk T, Brunkhorst FM, Spies C (2016). Hemophagocytic lymphohistiocytosis : a diagnostic challenge on the ICU. (Article in German). Anaesthesist.

[8] Zhou M, Li L, Zhang Q (2018). Clinical features and outcomes in secondary adult hemophagocytic lymphohistiocytosis. QJM.

[9] Kapoor S, Morgan CK, Siddique MA, Guntupalli KK (2018). Intensive care unit complications and outcomes of adult patients with hemophagocytic lymphohistiocytosis: a retrospective study of 16 cases. World J Crit Care Med.

[10] Guo Y, Bai Y, Gu L (2017). Clinical features and prognostic factors of adult secondary hemophagocytic syndrome: analysis of 47 cases. Medicine (Baltimore).

[11] Otrock ZK, Eby CS (2015). Clinical characteristics, prognostic factors, and outcomes of adult patients with hemophagocytic lymphohistiocytosis. Am J Hematol.

[12] Eloseily EM, Weiser P, Crayne CB (2020). Benefit of anakinra in treating pediatric secondary hemophagocytic lymphohistiocytosis. Arthritis Rheumatol.

[13] Shakoory B, Geerlinks A, Wilejto M (2022). Pos0339 Points to consider at the earliest stages of the diagnosis and management of hemophagocytic lymphohistiocytosis/macrophage activation syndrome (HLH/MAS). Ann Rheum Dis.

[14] Bami S, Vagrecha A, Soberman D (2020). The use of anakinra in the treatment of secondary hemophagocytic lymphohistiocytosis. Pediatr Blood Cancer.

[15] Ahmed A, Merrill SA, Alsawah F (2019). Ruxolitinib in adult patients with secondary haemophagocytic lymphohistiocytosis: an open-label, single-centre, pilot trial. Lancet Haematol.

[16] Wang Y, Huang W, Hu L (2015). Multicenter study of combination DEP regimen as a salvage therapy for adult refractory hemophagocytic lymphohistiocytosis. Blood.

[17] Zhang K, Jordan MB, Marsh RA (2011). Hypomorphic mutations in PRF1, MUNC13-4, and STXBP2 are associated with adult-onset familial HLH. Blood.

[18] Meng G, Feng S, Wang Y (2025). DEP regimen for the treatment of hemophagocytic lymphohistiocytosis: a review of published experience. Front Pharmacol.

[19] Grundy PE, Green DM, Dirks AC, Berendt AE, Breslow NE, Anderson JR, Dome JS (2012). Clinical significance of pulmonary nodules detected by CT and not CXR in patients treated for favorable histology Wilms tumor on national Wilms tumor studies-4 and -5: a report from the Children's Oncology Group. Pediatr Blood Cancer.

[20] Lehmberg K, Ehl S (2013). Diagnostic evaluation of patients with suspected haemophagocytic lymphohistiocytosis. Br J Haematol.

[21] Bergsten E, Horne A, Aricó M (2017). Confirmed efficacy of etoposide and dexamethasone in HLH treatment: long-term results of the cooperative HLH-2004 study. Blood.

[22] Gooptu M, Kim HT, Jacobsen E (2023). Favorable outcomes following allogeneic transplantation in adults with hemophagocytic lymphohistiocytosis. Blood Adv.

[23] Machowicz R, Suarez F, Wiktor-Jedrzejczak W (2022). Allogeneic hematopoietic stem cell transplantation for adult HLH: a retrospective study by the chronic malignancies and inborn errors working parties of EBMT. Bone Marrow Transplant.

[24] Kim H, Mizuno K, Masuda K (2024). A nationwide retrospective analysis of allogeneic hematopoietic stem cell transplantation for adult hemophagocytic lymphohistiocytosis. Transplant Cell Ther.

[25] Yoon JH, Park SS, Jeon YW (2019). Treatment outcomes and prognostic factors in adult patients with secondary hemophagocytic lymphohistiocytosis not associated with malignancy. Haematologica.

[26] Masood A, Wahab A, Iqbal Q, Davis J, Ehsan H, Hashmi H (2022). Efficacy and safety of allogeneic hematopoietic stem cell transplant in adults with hemophagocytic lymphohistiocytosis: a systematic review of literature. Bone Marrow Transplant.

[27] Meyer LK, Lee JC, Rocco JM, Nichols KE (2025). Histiocyte Society blueprint for hemophagocytic lymphohistiocytosis research: deciphering underlying disease mechanisms to optimize diagnosis and therapy. Haematologica.

[28] La Rosée P, Horne A, Hines M (2019). Recommendations for the management of hemophagocytic lymphohistiocytosis in adults. Blood.

[29] Griffin G, Shenoi S, Hughes GC (2020). Hemophagocytic lymphohistiocytosis: an update on pathogenesis, diagnosis, and therapy. Best Pract Res Clin Rheumatol.

[30] Jordan MB, Allen CE, Weitzman S, Filipovich AH, McClain KL (2011). How I treat hemophagocytic lymphohistiocytosis. Blood.

